# Drivers and Barriers of Breast Augmentation Surgery: Multinational Insights

**DOI:** 10.1093/asjof/ojaf117

**Published:** 2025-09-20

**Authors:** William P Adams, Francisco G Bravo, M Bradley Calobrace, Andre Cervantes, Caroline Glicksman, Craig Layt, Jie Luan, Lina Triana, Per Hedén

## Abstract

**Background:**

Although breast augmentation is generally associated with high levels of patient satisfaction, the number of women undergoing the procedure is low.

**Objectives:**

To evaluate global key drivers and barriers for women deciding to undergo breast augmentation.

**Methods:**

Qualitative semi-structured interviews were conducted with women aged 18 to 65 years to identify key themes regarding decision-making about breast augmentation. This was followed by a quantitative study with more in-depth analysis conducted in women who had the procedure, who were considering breast augmentation, or who were not considering breast augmentation.

**Results:**

In the qualitative interviews (*n* = 24), drivers of breast augmentation included feelings of dissatisfaction with breasts and wanting to improve confidence and self-esteem, whereas barriers included cost and safety concerns. For the quantitative study (*n* = 798 enrolled in the United States, United Kingdom, Brazil, and China), these themes were confirmed, with the strongest drivers being feeling more confident and deserving to feel satisfied with their breasts. For those women who had undergone or considered breast augmentation, there were an average of 6.5 reasons that were concerns or barriers to the procedure, including the safety of the procedure and implants, potential complications, and costs. These barriers were similar to those cited by participants who had not considered breast augmentation. The Net Promoter Score of participants who had undergone breast augmentation was 49, with 62% promoters.

**Conclusions:**

Given the mix of positive and negative drivers that impact women's decisions regarding breast augmentation, it is essential that physicians educate potential patients regarding surgical details, possible outcomes, and potential procedure-related complications.

**Level of Evidence:**

3 (Therapeutic) 
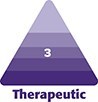

It is estimated that approximately one-half of adult women would prefer a larger breast size, as evidenced in the Breast Size Satisfaction Survey that included more than 18,000 women from 40 countries.^1^ Women are also motivated to undergo breast augmentation to improve their satisfaction with the overall shape and feel of their breasts.^2^ Consequently, breast augmentation is one of the leading cosmetic surgical procedures worldwide for women, with more than 1.8 million undergoing this procedure in 2023, and it is estimated that 35 million women worldwide currently have breast implants.^3,4^ A number of studies have shown that satisfaction with breasts significantly increases and is sustained following breast augmentation, alongside improvements in sexual and psychosocial well-being.^5,6^ However, despite overall patient satisfaction, the number of women having breast augmentation surgery has not increased in recent years.^3^

Perceptions of implant safety may affect potential patients' decisions regarding breast augmentation. Historically, the news media was the primary source of information regarding the safety and efficacy of breast implants. In December 1990, the television news program *Face to Face*, hosted by Connie Chung, aired on prime-time television. The episode introduced many Americans to issues surrounding breast implant safety and the regulatory process that controlled the manufacture and sale of silicone gel-filled breast implants in the United States. Negative media reports regarding the association between capsular contracture or rupture and silicone gel implants greatly impacted the use of these devices for many years, even though the scientific evidence supporting this association was of low quality.^7^ The regulation of silicone breast implants underwent a series of changes enacted by the Center for Devices and Radiological Health at the US FDA. This included the 1992 moratorium on the sale of silicone gel–filled breast implants in the United States and Canada that lasted from 1992 to 2006.^8^

Despite the unvalidated nature and potential inaccuracy of much of the information provided, digital media is an increasingly important source of information for women considering breast augmentation, as the vast majority of women now utilize the internet and social media platforms as their primary source of information regarding this procedure.^9^ Numerous online forums and groups are devoted to solely discussing negative opinions on breast implant–associated issues such as Systemic Symptoms Associated with Breast Implants (SSBI; also known as “breast implant illness [BII]”).^10^ SSBI is an umbrella term for a constellation of nonspecific systemic symptoms (most often “brain fog,” lack of motivation, fatigue, mood symptoms, joint pain, and hair loss) that patients who had breast augmentation with implants have reported as attributed to the procedure.^11^ It is also easy to access individual online stories and testimonies of patients who have undergone breast augmentation procedures and are not pleased with the results. On the other hand, stories abound of those who felt that they had experienced SSBI and had their implants removed. Some of these patients did not experience resolution of their symptoms, and others were eventually diagnosed with another medical condition and are now unhappy with the appearance of their breasts. These testimonials are seldom covered on any media platform. Outside the United States, other syndromes such as autoimmune syndrome induced by adjuvants (ASIA) and silicone incompatibility syndrome are based on anecdotal evidence and case reports that describe an association between silicone gel–filled breast implants and systemic symptoms; however, ASIA has not yet been accepted as scientifically valid by the rheumatology academic community.^12,13^

There have been several published studies documenting a reduction in self-reported systemic symptoms after breast implant removal.^14,15^ Notably, significant ongoing research, including work by groups such as McGuire et al, continues to explore this phenomenon.^14^ However, SSBI does not currently meet the criteria for a medical disease, with no specific symptoms that have been identified to date and no objective tests that are available.^16^ Moreover, recent scientific data support the role of anxiety and depressive disorders as underlying contributing factors to the problem.^17^ As a result, it is difficult to determine the prevalence of SSBI, as patients may be identified in clinical practice only when women request breast surgery revision or as members of specific SSBI online groups or forums.^10,11,18^

Breast implant–associated anaplastic large cell lymphoma (BIA-ALCL) is a rare complication of breast augmentation that occurs solely with textured breast implants.^19,20^ Starting in 2019, France's ANSM took action to ban certain types of textured breast implants, and other regulatory bodies took similar action.^21^ This resulted in Allergan's worldwide recall of their BIOCELL textured breast implants and tissue expanders. In October 2021, the US FDA issued a boxed warning for all breast implants, mandating that patients be informed of the significant risks associated with breast implants:^22^ “Breast implants have been associated with the development of a cancer of the immune system called breast implant–associated anaplastic large cell lymphoma (BIA-ALCL). This cancer occurs more commonly in patients with textured breast implants than smooth implants, although rates are not well defined. Some patients have died from BIA-ALCL.” Following the introduction of the boxed warning, many women have been less likely to undergo breast augmentation due to concerns regarding BIA-ALCL.^23,24^ Breast implant–associated squamous cell carcinoma (BIA-SCC) is a separate rare complication of breast augmentation.^25^ To date, the FDA has gathered information on 19 cases of BIA-SCC from the literature and has published recommendations for both patients and health care providers.^26^ Chronic inflammation is believed to be a driver of the process.^27^

Other concerns of women considering breast augmentation are around the procedure itself (eg, the potential need for general anesthesia, recovery process, pain) and local complications (eg, infection, implant rupture, scarring).^6,24^

Given the recent developments described above, including discussions around SSBI and BIA-ALCL, as well as the introduction of new products and advancements in minimally invasive procedures, we conducted 2 studies to better understand the current sentiment around breast augmentation. The objectives of the current studies were to identify the drivers and barriers that influence women's decisions about whether to undergo breast augmentation surgery and to characterize their patient journey. The current investigation began with a qualitative interview study, the results of which informed a subsequent quantitative survey study.

## METHODS

### Qualitative Study

The objective of the qualitative study was to gain a better understanding of the breast augmentation patient journey, including initiating factors, influences, and barriers to uptake.

Women from the United States and the United Kingdom were recruited into the qualitative study between November 2019 and January 2020. Eligible participants included women aged 18 to 65 years who had undergone breast augmentation, those who had considered augmentation and had or had not attended a consultation with a health care provider, those who had not previously considered breast augmentation, and those who had undergone lumpectomy due to breast cancer.

Sixty-minute, web-enabled, semi-structured interviews were conducted by a market research company employed by the sponsor to cover a selection of topics, which included the following: (1) the patient's surgical and nonsurgical procedure history and their feelings toward cosmetic surgery in general; (2) their perceptions of themselves in terms of their best and worst qualities, self-confidence, and any insecurities they might have; (3) previous considerations of breast augmentation surgery and which factors might promote or prevent action; (4) their thoughts and emotions leading up to a consultation regarding breast augmentation and the subsequent actions taken, if any; (5) awareness and knowledge of the breast implant procedure and implant types (awareness and opinions of individual breast implant manufacturers, experience of the procedure and recovery in those who underwent breast augmentation, knowledge regarding safety issues concerning breast implants and its impact on their decision-making); and (6) reflection on their decision regarding breast augmentation and ranking of drivers and barriers.

Demographic information was also collected at the time of the interview, and participants received a small gratuity for their participation in the study.

### Quantitative Study

The objective of the quantitative study was to further determine the most important triggers, drivers, and barriers to breast augmentation/reconstruction from the initial consideration to the final decision. These were based on the findings from the qualitative study.

Women considering or who had undergone breast augmentation were recruited from the United States (aged 18-54 years), United Kingdom (aged 18-54 years), China (aged 18-45 years), and Brazil (aged 18-45 years). Women who had not considered breast augmentation were also enrolled. Additionally, women in the United States aged 18-65 years who had a mastectomy or lumpectomy following breast cancer diagnosis were recruited.

Participants completed a 25-min online survey between February and March 2020, conducted by the same market research company as above. Participants were first asked about their history, consideration, and health care provider consultation of breast augmentation (or reconstruction for patients with a diagnosis of breast cancer). They were presented with lists of reasons for considering breast augmentation/reconstruction—covering the domains of physical factors, external influencers, and emotional influencers—and were asked to rank their importance. Specific outcomes following breast augmentation/reconstruction and main concerns prior to the procedure were also explored using predefined lists, with participants ranking their top 3 reasons. Participants were asked which sources of initial information regarding the breast augmentation/reconstruction procedure they used and what types of information they looked for.

Participants who had undergone breast augmentation/reconstruction were asked about their experiences regarding satisfaction with the outcomes and any complications (rated from 1 [much more difficult than expected] to 7 [much easier than expected]). Lastly, the Net Promoter Score (NPS), which has previously been used in health care studies, was calculated.^28^ Participants were asked how likely they would be to recommend breast augmentation on a Likert scale of 1 to 10; promoters were defined as women who scored 9 to 10, detractors as those who scored 0 to 6, and passives as those who scored 7 to 8. NPS is calculated by subtracting the percentage of detractors from the percentage of promoters.^28^

## RESULTS

### Qualitative Study

A total of 24 women participated in the qualitative study, 11 from the United States (aged 22-64 years) and 13 from the United Kingdom (aged 24-48 years). Of these, 8 had undergone breast augmentation, 10 had considered augmentation, 4 had not considered augmentation, and 2 had a lumpectomy following breast cancer.

A summary of key themes is presented in Supplemental Table 1. There appeared to be a growing acceptance of plastic surgery in general, although the participants had mixed opinions and perceptions about how it should be approached—the key theme was that many emphasized the importance of looking natural following cosmetic procedures rather than becoming unrecognizable. Regardless of personal opinion, participants were strongly protective of their individual freedom to decide how to change their appearance.

Initial drivers for considering breast augmentation included deep-seated feelings of dissatisfaction with their own breasts. For some participants, nursing or raising children were the most influential factors in their decision-making journey. General societal perceptions and personal relationship dynamics also played a significant role in deterring or motivating the decision to pursue breast augmentation. Some participants indicated that they would mull over the decision to undergo breast augmentation surgery for many years until something triggers them to take further action.

The findings support that women gather preliminary information about breast augmentation through online channels, forums, and images, which can either strengthen or weaken the desire to arrange a professional consultation. The survey also indicates that many women are nervous during consultations and are looking for a professional confirmation that breast augmentation will be safe and achieve successful results.

The participants who had undergone breast augmentation stated feelings of excitement as they approached the date of their operation and reported having an overall positive experience from surgery through recovery. In general, participants who had undergone breast augmentation surgery felt self-assured and pleased with their decision, reporting improved levels of confidence that may be life-changing.

Participants who had considered breast augmentation surgery may have postponed or changed their decision due to personal obstacles or concerns about the procedure; learning of the potential risks or adverse outcomes may also have influenced their decision-making. Those participants who had rejected breast augmentation surgery were opposed to artificial physical enhancements due to safety or personal principles. Stated key barriers to pursuing breast augmentation included cost, concerns about complications, and timing.

Of the participants who had breast cancer, responses indicated that managing their disease leads to a considerable amount of physical and emotional stress. Although they may have considered implants, lack of insurance coverage, limitations, or excessive copayments may have prevented them from going through with the procedure.

### Quantitative Study

**Figure 1.**
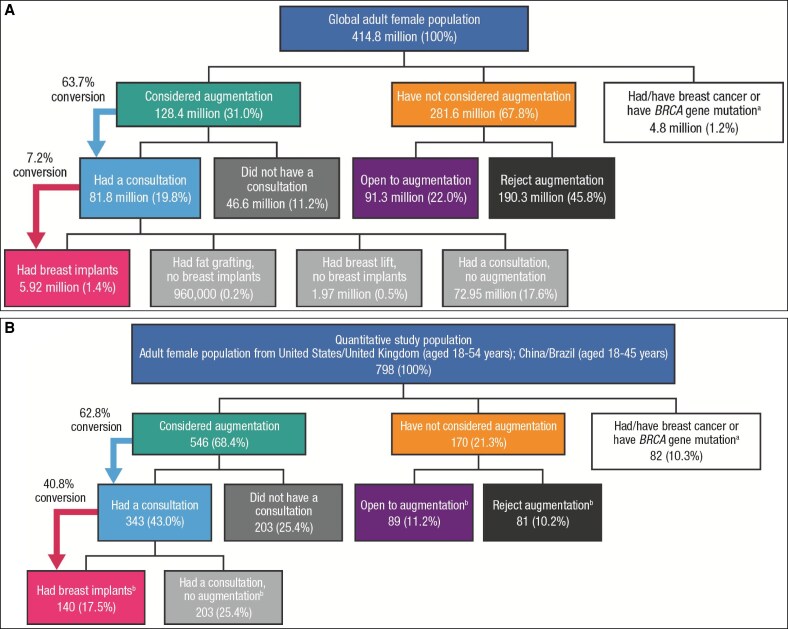
Penetration of breast augmentation surgery (A) globally (*n* = 414.8 million) and (B) in the quantitative study population from the United States, United Kingdom, China, and Brazil (*n* = 798). ^a^An assumption was made that 1 in 400 (0.25%) women have a *BRCA* gene mutation (Source: National Breast Cancer Foundation, Inc. https://www.nationalbreastcancer.org/what-is-brca/). The sample was balanced and weighted to reflect census data for age (base: market sizing sample, *n* = 6358). ^b^Percentages may not sum exactly due to rounding. *BRCA*, BReast CAncer gene.

A total of 414.8 million adult women made up the global female population during the market research study period (Figure 1A), of which 798 women across 4 countries participated in the quantitative study (Figure 1B and Supplemental Table 2). Of these, 140 (17.5%) had undergone breast augmentation surgery (Figure 1B), and a further 406 (50.9%) had considered breast augmentation but had not undergone the procedure. The mean age of these participants ranged from 31 to 34 years across the different countries. One hundred and seventy participants (21.3%) had not considered breast augmentation (81 completely rejected and 89 were open to the idea). Of the 82 participants (10.3%) who had a diagnosis of breast cancer, 31 had implant surgery. Globally, of those women who had a consultation regarding breast augmentation, only 7.2% proceeded with the surgery, with an overall penetration rate of 1.4%—defined as the proportion of the global adult female population who have undergone the procedure (Figure 1A).

**Figure 2.**
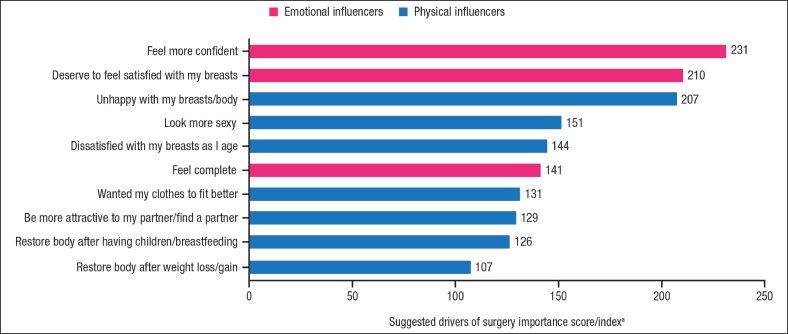
Most frequently cited drivers of breast augmentation surgery among women who had undergone the procedure or were considering the procedure (*n* = 546). ^a^Drivers with a score of >100 are of above-average importance.

**Figure 3.**
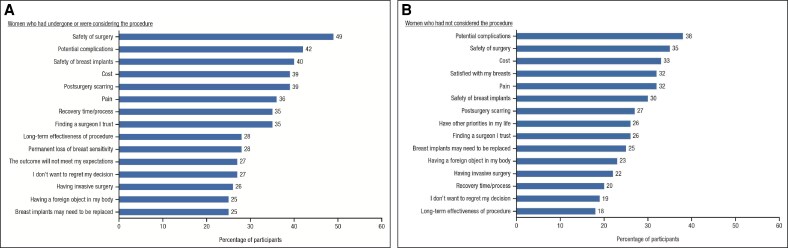
Most frequently cited barriers to breast augmentation surgery among women who (A) had undergone the procedure or were considering the procedure (*n* = 546), and (B) who had not considered the procedure (*n* = 170).

Among the 546 participants who had undergone or considered breast augmentation surgery, the most frequent suggested drivers for having the procedure were to feel more confident (42.3%), deserving feelings of satisfaction with their breasts (38.5%), and unhappiness with their breasts/body (37.9%; Figure 2 and Supplemental Figures 1, 2). These were similar across the 4 countries, with the exception of China, where the top 3 drivers were feeling more confident, unhappiness with their breasts/body, and to look sexier. The greatest unprompted emotional driver was feeling more confident/having improved self-esteem, cited by 13% of participants who had undergone breast augmentation surgery and 5% of participants who had considered breast augmentation but had not yet made a decision. Among participants who had not considered breast augmentation, the highest frequency of unprompted responses regarding others' rationale for surgery was to look more attractive (26%) and to feel more confident/improve self-esteem (33%). For those participants who had undergone or considered the procedure, there were an average of 6.5 reasons that were concerns or barriers to breast augmentation surgery, with the most often cited being safety concerns regarding the surgery (the most common factor in China and Brazil) or the implants, potential complications, cost (the most common factor in the United States and United Kingdom), and scarring (Figure 3). Similar concerns and barriers were also cited by participants who had not considered breast augmentation surgery, with an average of 5.3 reasons.

Concerns regarding potential rupture of silicone gel–filled breast implants (ranked as the highest concern in all 4 countries) and BIA-ALCL (Table 1) were similar between participants who had undergone or had considered breast augmentation compared with those who had not considered the procedure. However, more participants who had undergone or were considering breast augmentation surgery were concerned about SSBI/BII than those who had not considered breast augmentation (37% vs 25%, respectively). Twice as many participants who had not considered breast augmentation surgery stated that they had insufficient knowledge about breast implant safety as those who had undergone or were considering the procedure (26% vs 13%, respectively).

**Table 1.** Safety Concerns Regarding Breast Implants Among All Participants From the Quantitative Study

Concern	Women who had undergone or considered breast augmentation surgery (*n* = 546), %	Women who had not considered breast augmentation surgery (*n* = 170), %
Possible rupture of silicone gel–filled implants	56	54
BIA-ALCL	38	32
SSBI/BII	37	25
Possible deflation of saline-filled breast implants	31	25
The silicone shell and/or silicone gel–filled implants	29	22
The surface texture of implants	28	14
Not enough knowledge about the safety of implants	13	26
I have no concerns about the safety of breast implants	7	3

BIA-ALCL, breast implant–associated anaplastic large cell lymphoma; BII, breast implant illness; SSBI, systemic symptoms associated with breast implants.

**Figure 4.**
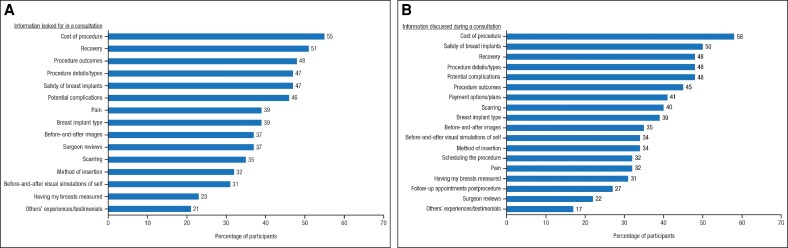
Alignment of information needs with topics discussed during consultations concerning breast augmentation surgery (*n* = 343): (A) information participants looked for in a consultation, and (B) information participants discussed during a consultation.

**Figure 5.**
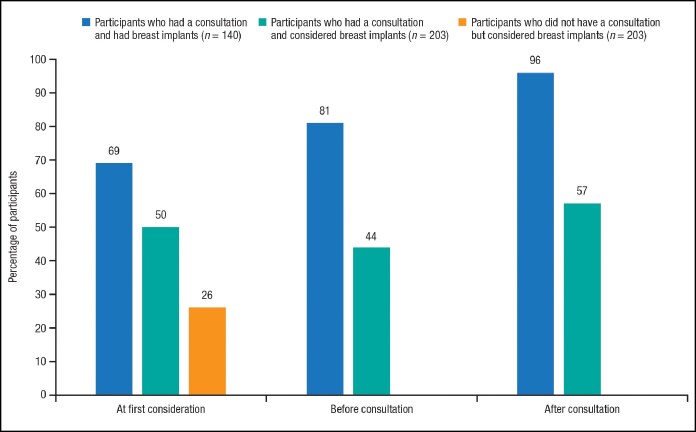
Breast augmentation decision certainty before and after cosmetic specialist/surgeon consultation. Data shown are women who are certain or very certain about undergoing breast augmentation.

With respect to consultations regarding breast augmentation surgery, the topics discussed were broadly in line with the information the participants looked for during a consultation, most often procedure cost, breast implant safety, recovery, details of the procedure, potential complications, and outcomes (Figure 4). Notably, there were no discussions of the emotional reasons women consider breast augmentation, as identified in the women who had undergone breast augmentation surgery. Overall, the certainty of participants regarding breast augmentation increased following consultation (Figure 5 and Supplemental Figure 3). Among participants considering breast augmentation surgery who did not follow through with a consultation, the most frequently cited barriers to consultation were cost (55%), needing more time for consideration (46%), deciding not to proceed at that time (27%), ongoing research about the procedure (26%), and looking for the right cosmetic specialist/surgeon (22%).

Among participants who had undergone or had considered breast augmentation, actions when first considering the procedure included searching for more information (65%; the most frequent response in participants from all 4 countries), looking for “before” and “after” photos (51%; second most frequent response in participants from the United States, United Kingdom, and Brazil), talking to close family/friends (45%), searching for cosmetic surgeons (42%; the second most frequent response in participants from China), and talking to others who had undergone breast augmentation surgery (40%). The most common sources of information included general internet searches (59%; the most common response in all 4 countries), surgeon/clinic websites (39%), friends or family (37%), and direct contact with a surgeon/clinic (35%).

Overall, for participants who had undergone breast augmentation surgery, patients generally directed the choice of augmentation procedure, whereas physicians selected the specific brand of implant used (Supplemental Figure 4). Among those who had surgery, 53% felt that the procedure was easier or much easier than they had expected, and 50% felt that postprocedure recovery was easier or much easier than expected (from between 34% of responses in participants from the United States to 70% of responses in participants from Brazil). A total of 62% of participants who had undergone breast augmentation surgery and 34% of participants who had considered breast augmentation surgery would promote the procedure to others, compared with 13% of those who had not considered breast augmentation surgery. The NPS was 49 in women who had undergone breast augmentation surgery (including 62% promoters). Conversely, 73% of women who had not considered breast augmentation surgery would actively advocate against it (NPS of −60) compared with 37% of women who had considered the procedure (NPS of −3) and 13% of women who had undergone the procedure.

## DISCUSSION

Key drivers of breast augmentation surgery could be divided into positives (wanting to increase personal satisfaction with their body, enhanced self-esteem/confidence) and negatives (unhappiness with current body/breasts, aging-related issues). Regarding the positives, studies have shown that breast augmentation can enhance self-esteem, as breasts are often considered an important facet of a woman's identity and her feelings of femininity.^29^ With respect to the negatives, the findings of the current study are in broad agreement with a previous study in which respondents cited poor body/breast image, reduced self-esteem, and impact on intimate relationships as motivators for seeking breast augmentation surgery.^30^ These results indicate that the underlying motivation for patients seeking breast augmentation is most often psychological or medical. However, during consultations, the majority of these factors were not discussed. Doing so could potentially improve the patient experience; conversely, the surgeon performing the procedure may not be equipped to explore topics that are outside of the functional aspects of the surgery itself. The NPS of participants who had undergone breast augmentation was notably high (49 from a possible highest score of 100), including 62% promoters, indicating a high level of satisfaction. For comparison, customers who experience the Ritz-Carlton included 63% promoters with an NPS of 39.^31^ Key strategies to enhance patient satisfaction and engagement with breast augmentation include advancements in implant technologies, surgical techniques, and preoperative processes (eg, breast analysis and markings), which could benefit from incorporating objective, mathematical frameworks or simulations alongside clinical expertise.^32^ Providing breast surgeons with additional educational opportunities on the latest developments and tools will be instrumental in achieving these goals.

Despite these high levels of patient satisfaction, there is still a considerable population of women who have considered breast augmentation surgery, but who have not continued the process with a consultation or procedure. Only 7.2% of women proceed to surgery after a consultation, resulting in only a 1.4% global penetration rate, thus highlighting the need for better engagement and clearer communication. The initial consultation is a key point in the patient's journey. A generation ago, patients received much of their information from friends and family members prior to consultation and depended upon the office visit to learn about the procedure in detail and its associated risks and benefits from the surgeon. However, with the greater use of online resources, patients may now arrive at the consultation with preconceived ideas of precisely the procedure they need and expectations of the surgical outcomes. These may be influenced by online “before” and “after” images that may have been edited or filtered, which can lead to unrealistic expectations. Indeed, in the current study, the first action taken by a total of 65% of women was searching for information, 51% looked for photos of breast augmentation results, and 45% discussed the procedure with someone they know. To address misconceptions and bridge the gap between interest and action, testimonials from highly satisfied patients and data-driven campaigns could highlight quality-of-life improvements achieved post-surgery. Initiatives to collect and share real-world outcomes could amplify awareness and encourage more patients to proceed with consultations or the procedure.

Over the past decade, there has been a downward trend in general internet searches (eg, Google) regarding breast augmentation.^33^ This may be, in part, explained by a switch to other platforms (eg, Facebook, Instagram, YouTube, and TikTok), which contain readily accessible visual materials of personal experiences and allow virtual “word of mouth” spread of information.^34^ However, the validity of information provided via these platforms may be questionable, and there is typically great bias and a lack of balance. Furthermore, another aspect of digital/social media is the potential for negative opinions from public figures or celebrities who have undergone breast augmentation. This can engender feelings of anxiety that breast augmentation will result in unnatural-looking breasts or long-term health issues.

Google trends data have shown increased activity in searching for SSBI, BIA-ALCL, and implant removal, and peaks in such search activity have often occurred following newspaper reports, celebrity stories, and FDA safety warnings.^10,33,35^ More specifically, this has been accompanied by a rise in social media groups and forums focused on implant safety concerns, most notably SSBI.^10^ It is known that some patients believe they have developed SSBI even after successful clinical outcomes of breast augmentation and in the absence of physician evaluation.^16,36^ This may be a consequence of “cyberchondria”—that is, an increase in anxiety about an individual's health status resulting from excessive and compulsive reviews of online health information.^37^ Studies have also documented that symptom reporting similar to those of SSBI/BII may occur with high frequency in females without breast implants and are very common and strongly associated with health care visits.^34,38^ Self-reported systemic symptoms may be caused by anxiety disorders, medication side effects, menopause, and other age-related changes that are unrelated to breast implants.^39,40^

Concurrently, there has been an upward trend in recent years for the removal of breast implants, with more than 41,000 of these procedures reported in the United States in 2023.^41^ There are multiple variables that influence a woman's decision to remove her implants without replacement. These include aging patients, aging implants, weight gain, and a trend for smaller breasts.^42,43^ However, breast implant removal can be costly, may involve complications (including those related to the surgical procedure itself), and may result in undesirable esthetic outcomes.^44^ Notably, knowledge of SSBI and BIA-ALCL among women attending breast augmentation consultations varies widely geographically.

There has also been a rapid increase in the past few years in the number of peer-reviewed publications concerning adverse outcomes associated with breast implants.^10^ However, this is inconsistent with the rarity of BIA-ALCL and BIA-SCC in light of the improvements over the last 2 decades that have optimized patient outcomes and achieved great reductions in postprocedure complications.^20,25,45,46^

Concerns about the safety of the surgical procedure and breast implants themselves accounted for the majority of barriers to breast augmentation surgery, including risk of cancer, poor cosmetic outcomes, and the longevity of the implants. Women may be concerned about the surgical procedure itself, including anesthesia and the recovery process, and the small number of deaths among cosmetic surgery patients reported in the media may be uppermost in their minds. Fear of an inadequate esthetic result (especially the importance of looking natural rather than becoming unrecognizable), concerns regarding safety, and worry with respect to the recovery process/time to recovery have recently been identified as 3 important patient-related outcome measures associated with the success of esthetic plastic surgery.^47^ With respect to safety, fear of general anesthesia is a particular concern for many patients.^48^ Esthetic surgeons should play an active role in addressing or resolving such barriers. A major part of this endeavor is further research and development of devices and techniques to reduce the risk of complications and increase patient safety, together with greater education and improved training of breast surgeons.

Surgeons and other health care professionals may also need to challenge any inaccurate or misleading information patients have gathered and ensure thorough probing and discussion on why the procedure a patient has predetermined as appropriate may not be the right one for them. Indeed, patients may not consider their own unique situation or limitations, which can impact final outcomes. Dedicated patient educators can provide accurate and appropriate information to patients to guide their decision-making and assist surgeons with encouraging realistic expectations for the procedure outcome.^49^

As with all surveys, the current study includes some limitations. This study collected data before and during the COVID-19 pandemic, when many people were perhaps more concerned about their general health than usual, and the consideration for cosmetic enhancements may have been less of a priority. The qualitative study centered on 2 countries with similar female populations and might not reflect behaviors and social drivers of breast augmentation in other regions (eg, Latin America and Asia). However, the quantitative study was conducted to include participants from the United States, United Kingdom, Brazil, and China. Indeed, a recent survey found women in 5 East Asian countries shared similar trends in rates of breast augmentation and willingness for future breast augmentation.^50^ However, many regions were not included in the current study (eg, Middle East and North Africa, South Asia, or Continental Europe) that represent a significant share of the global population, and may have striking cultural differences from the studied regions. As such, overall trends identified in the current study may not fully reflect global attitudes or the situation in all countries. The inclusion/exclusion criteria may also have influenced the observed outcomes, due to the younger age limit in Brazil and China (45 years) compared with the United States and the United Kingdom (54 years).

## CONCLUSIONS

In both the qualitative and quantitative studies presented, for women who had breast augmentation surgery and for those who had considered the procedure, the top 3 drivers of surgery were to feel more confident, because the patient deserved to feel satisfied with their breasts, and to resolve their unhappiness with their breasts/body. Safety-related concerns (including the safety of surgery, potential complications, and the safety of breast implants) were the most commonly reported barriers to undergoing the procedure among those who were considering breast augmentation surgery. Given the challenging decision faced by patients considering breast augmentation surgery, it is essential that physicians, plastic surgery societies, and industry professionals provide clear and balanced real-world information about the benefits and risks of the procedure. Doing so may help to counter some of the unrealistic and misleading information that is pervasive in current online sources.

## Supplemental Material

This article contains supplemental material located online at https://doi.org/10.1093/asjof/ojaf117.

## Supplementary Material

ojaf117_Supplementary_Data
