# Supplementary material for: Drivers and Barriers of Breast Augmentation Surgery: Multinational Insights
Source: Aesthet Surg J Open Forum. 2025 Sep 20;7:ojaf117. doi: 10.1093/asjof/ojaf117 (PMC12709286; doi:10.1093/asjof/ojaf117)
Supplement: ojaf117_Supplementary_Data [file ojaf117_supplementary_data.docx]

**Supplemental Table 1. Summary of key themes from the qualitative study**

| Interview topic | Key themes | Examples |
| --- | --- | --- |
| Feelings about plastic  surgery overall | - Looking natural and maintaining   true features   - Disapproval of changing appearance to the point where their original features   are unrecognizable   - Positive attitude towards numerous minor adjustments to preserve a youthful and natural appearance or to correct   self-perceived flaws in order to boost their  self-confidence   - Disapproval of cosmetic surgery due to safety risks, costs, and beliefs that women should be happy with the way they look or how they age naturally | “*There's a difference between just something small and wanting to feel confident about yourself…that’s fine. But then you've got these people that just…have to change every single thing because you want to be the prettiest, the best…I think there's a line and it's easily crossed.”*  *“I'm for it. I don't think that you have to go excessive to achieve results. Things have gotten better and better over the years…Society is getting better, but there still is a little bit of a stigma…They still are a little uncomfortable with it.”* |
| Feelings about breast augmentation | | |
| Women who have had breast augmentation surgery | - It is a personal choice made on their own accord, and for their own personal benefit - They may not be open about their augmentation with others because they expect to be judged negatively, or feel it is a private matter - The influence of friends and personal stories plays a powerful role in driving the decision to follow through with the procedure - In general, there is great satisfaction with the outcomes of the procedure and report greater levels of confidence after surgery | *“I definitely think it has been a big change. I'm a big advocate for women who want to do this. I think it's a great thing to do…because it has changed my life.”*  *“I love it. I feel more confident. I paid for it on my own. I saved up and earned something I wanted for so long. I feel fulfilled. I feel like I look better. Overall, I feel it's very empowering.”* |
| Women who have considered breast augmentation surgery | - For women yet to make a decision, the principle of surgery is generally not rejected, but is being postponed due to “life issues” getting in the way (eg, recovery time strains professional or family obligations) - Those who are in the stage of researching may become unsure about implants after learning about the risks, or adverse outcomes - The ability to finance the surgery is also an obstacle | *“I got a lot of information and I thought about it and talked to my husband about it. It wasn't feasible for our schedules, at that point.”*  *“I know that you have to take quite a lot of time out of the workplace. I didn't want to do that.”*  *“A couple of the girls I know…The experiences that they have scared me more away—just not wanting to have to do multiples or repairs, and being down for a long time.”* |
| Women who rejected breast augmentation surgery | - Disapprove of invasively implanting unnatural materials into their body, unless it is for necessary medical reasons - Concerns around the surgery are considerable and go beyond the implant itself—women in this group focus on the general risks that a major surgery involves, as well as its recovery period and subsequent scarring - Strong moral principles (eg, the body is a “temple”) | “*We're all different, and I think it's not natural to alter yourself…I know society loves surgical procedures and tattoos, piercings and all that stuff. I'm not into any of that...Our bodies are sacred and they're just not meant to be altered like that, unless there is some real surgical or medical need for a procedure.”*  *“I just feel like it's a foreign object in your body. There's no way to alter it if something goes wrong. You have to get cut open all over again*.” |
| Initial drivers for considering breast augmentation surgery during early development | - Women compare their breasts to other women’s from an early age - Teasing from peers or family during youth leads them to feel unattractive or less feminine and furthers insecurity - If their breasts do not mature as they would like, or if they are perceived to be awkward and unflattering through adulthood, they may have a stronger propensity for exploring surgical solutions | *“I was made fun of….I didn't have any boobs…they called me mosquito bites. Then it went from mosquito bites to the ‘itty-bitty titty’ committee...from the time I was young, all the way up, I was always put down by it and made fun of…A lot of it came from family…I have lots of sisters and they're all very well endowed.”* |
| Influencers in the decision-making journey | | |
| Before nursing | - Some women may have the misconception that implants can impair their natural ability to nurse children. Uncertainty surrounding changes to the breast tissue and mammary functioning can ultimately discourage them from proceeding with surgery until they are finished with childbearing altogether | *“I was afraid that I wanted more kids because I was in my 20s...That kept me from getting them (sooner) because I wanted to breastfeed.”* |
| After nursing | - Mothers who are finished nursing feel compelled to lift or restore the shape of   their breasts   - Some may be more emotionally restrained by their will to set a positive example for their daughters, or because of guilt based on the perception that breast augmentation is   self-centered | *“The reason that I haven't committed to a surgery at this point is because my daughter is 16. She was a teenager when I first considered it…Then I saw her looking at herself in the mirror…I don't want to give her any body image issues to have to think about. I don't want her to look at her own body and not be happy with what she sees. We're waiting until she's a little bit older…I want to make sure that I'm a good and healthy role model for her body image.”* |
| Social stigma | - Several women believe that society stigmatizes women who get breast implants for being “fake,” “shallow,” or “trashy” - Although a few think that younger generations are more accepting, inhibitions related to stigma are shared by both younger and older women. They may be particularly apprehensive or anxious about being   judged negatively | *“I think I'm quite judgmental, actually. That's probably stopping me from doing it…I think men like looking at fake breasts, but they don't really want to date someone with fake breasts…It’s not authentic. Everyone says the Kardashians are so plastic. We make fun of these people for having so much surgery. It's hypocritical if we get it done, too.”* |
| Interpersonal relationship dynamics | - Most partners are supportive of the choice that would make them happy, and rate their partner’s influence on the decision to be less than 50% - Romantic partners may be negative influencers in the decision-making process by inspiring natural self-acceptance instead of augmentation, or raising concerns about the risks of surgery | *“My husband has made me watch a lot of Botched episodes. That has swayed me a little bit. He's like, ‘Please don't ever do anything. Look what happens.’ I’m terrified to think of what could go wrong when it's unnecessary.”* |
| Motivators of breast augmentation | - Getting recommendations or advice from other women who have gotten a breast augmentation can inspire undecided considerers to finally move forward after deliberating the procedure for years - Having enough money to spend on   the procedure   - Seeing a friend who gets implants and has good results - Significant weight loss or gastric   band surgery   - Receiving recommendations for a trustworthy surgeon - Finishing child rearing - Weightlifting or body building | *“It was always on my mind that I wasn't happy with them, but…what triggered me to go and look was—I saw a friend from school…she was in the same boat as me when we were in school, she was really flat chested....She'd had them done about eight weeks prior to me seeing her. I couldn't believe the difference. She said she was so happy with them, and it was the best thing that she's done. That's what got me started to go and research it.”* |
| The impact of breast cancer | - Upon learning that they need a lumpectomy, women’s primary concern is to treat the cancer and preserve their health - Patients may not be eager to undergo an invasive cosmetic operation, even if it promises an emotional end benefit - Women who have had a lumpectomy report being unhappy with the appearance of their breasts and feeling uncomfortable   with themselves   - Even if breast reconstruction is suggested by their surgeon early on, some do not immediately proceed to get implants because of its potential for exacerbating their current physical, mental, or financial burden - A few may be advised to wait until their breasts heal from the lumpectomy before considering breast augmentation, since the breast tissue is prone to change as it heals - Women are open to considering reconstructive implants in the future if the surgery would be covered by their insurance | *“I was still going through a mix of emotions from finding out I had breast cancer, having to have surgery, going through chemotherapy, then going through radiation—all that takes a toll on your body. It was both physical and mental…I felt like my mental capacity could not hold another procedure. I just needed to regroup all the way around.”* |
| Barriers to breast augmentation | | |
| Cost | - Few have investigated the true cost of breast augmentation or the ways in which the procedure can be paid for (eg, loans or payment plans) - Most expect to pay at least $5000 up front and believe they cannot afford the surgery. They are also pre-emptively concerned about the cost of paying for future complications or “touch up” procedures - Some wonder whether they would be sacrificing quality and safety if opting for a lower-cost surgeon or clinic | *“The financial thing for me is always going to be the downside, because it is so expensive. And it is a huge decision for me to make…I'm not from a wealthy family….I’ve worked for this amount of money. What am I spending on?”* |
| Future complications | - Many women worry about noted risks and safety concerns, such as severe scarring, leakage, or rupture. These risks can be highly off-putting to those who are on the fence about their decision - Photos and stories of negative outcomes, financial expenses they may incur as a result of future complications, or corrective procedures if their initial results   are unsatisfactory | *“You hear about people having them removed, or that there was leaking or complications…You just don't know the potential complications in the future.”* |
| Timing | - Obstacles in their life circumstances can impede on the timing for surgery and subsequent recovery - They may feel that it’s never the right time to undergo the procedure, especially if they are working or if they have not finished childbearing and nursing - Some may also be apprehensive if their body is prone to weight fluctuation, which could also alter their breasts and surrounding tissue | *“I convinced myself that recovery and scheduling would be too big of a conflict to overcome. I received information from a surgeon but never committed because I had decided to go back to school full time…It was never the right time.”* |
| Considerations prior to consultation | | |
| Those who arrange a consultation | - Tend to be excited after learning about positive outcomes - Want a thorough and informed explanation that is tailored to their specific needs   and goals   - Acknowledge that consultations are free and necessary for establishing accurate expectations - Can easily access specialists that are highly rated online or those recommended by trusted friends or family | *“I think it's important to know a little bit about how the procedure is done, and potential risks and things like that, but…I'd rather speak to the surgeon that's actually going to do the procedure to get more of that information.”* |
| Those who do not arrange a consultation | - Tend to be discouraged by the information they have seen online or heard from other women’s experiences - May be fearful of the physical and financial investment and are more likely to question whether the perceived emotional benefits outweigh the risks and cost - May not have access to a good specialist in close proximity | *“I think I was quite discouraged by the fact that you have to quite frequently have them done every 10 or 12 or however many years that it's necessary. I know there's not an expiration on implants, but having that ongoing procedure. That discouraged me…”* |
| Consultation | | |
| Number of consultations | - Women tend to have multiple consultations before making their decision - Even if they are satisfied with their first consultation, many believe it is important to have a second or third opinion before choosing to undergo breast augmentation surgery. Some feel more secure about their choice when they know they have more than one option |  |
| Positive influencers during consultation | - Trustworthy surgeons that provide honest and practical expectations without   being “pushy”   - Seeing compelling before-and-after photos of the surgeon’s previous work - Learning more about the implant types and different methods for insertion - Attentive clinic staff that reduce anxiety surrounding the surgical process | *“When initially you go in there, obviously you're a bit reserved, don't really know what to expect. But because he was so sure of himself and what he was saying, and I also felt like it wasn't really a sales pitch…He just wasn't pushy at all, and I felt like I was talking to a doctor. He was asking about my medical history, if I exercise, and different life events and things like that. It really felt like he knew what he was talking about. That relaxed me.”* |
| Perioperative expectations and experiences | - Although most are enthusiastic and feel   well prepared for the procedure, some may be nervous about the surgery itself or experiencing pain post-operation   - Several women remark that their recovery process was “smooth” and that they were only somewhat affected by pain and discomfort after surgery. Some describe it as a “pulling” sensation in their chest muscles and say it did not restrict their ability to otherwise function normally | *“I was really, really excited. It was almost like when you're a child and it's the night before Christmas, and you couldn't sleep. It was a little bit like that. I was very nervous because I've never had any kind of surgery. I've never been put to sleep or anything like that. I know of course these days there's very minimal risk with things like that. The only thing I was scared about was being put to sleep. That was the only thing.”*  *“It wasn't pain at all…People say it hurts more to get it underneath the muscle because it's a bit more invasive, but it just felt like I had pulled a chest muscle at the gym. It didn't feel too bad. After that, within day or two…I was able to get up and make some light dinner…It feels a bit tight, so you try and avoid having to pick anything up, really. Apart from that, you can get up, you can go to the toilet, you can literally move about as you would.”* |

**Supplemental Table 2. Demographic characteristics of the quantitative study participants**

|  | Had implants | Considered augmentation | Have not considered augmentation |
| --- | --- | --- | --- |
| United States | n = 50 | n = 100 | n = 56 |
| Age, years |  |  |  |
| Mean | 43 | 34 | 37 |
| 18-29, % | 6 | 35 | 31 |
| 30-39, % | 26 | 40 | 27 |
| ≥40, % | 68 | 25 | 42 |
| Ethnicity, % |  |  |  |
| African American or Black | 2 | 8 | 10 |
| Asian or Pacific Islander | 8 | 2 | 13 |
| Caucasian or White | 94 | 84 | 68 |
| Native American | 0 | 2 | 3 |
| Hispanic or Latino | 6 | 4 | 12 |
| Marital status, % |  |  |  |
| Married | 56 | 63 | 43 |
| Single, never married | 12 | 26 | 41 |
| Widowed/divorced/separated | 30 | 11 | 13 |
| People in household |  |  |  |
| Mean | 2.6 | 3.2 | 2.7 |
| Percentage with children aged <18 years | 48 | 51 | 38 |
| Employed, % | 60 | 61 | 47 |
| College level education and above, % | 40 | 34 | 41 |
| Mean annual income, $US | 78,000 | 78,000 | 50,000 |
| Area of residence, % |  |  |  |
| Urban/city | 24 | 24 | 21 |
| Suburban | 56 | 34 | 55 |
| Rural | 20 | 39 | 24 |
| United Kingdom | n = 30 | n = 100 | n = 39 |
| Age, years |  |  |  |
| Mean | 36 | 33 | 37 |
| 18-29, % | 27 | 34 | 26 |
| 30-39, % | 40 | 48 | 30 |
| ≥40, % | 33 | 18 | 44 |
| Ethnicity, % |  |  |  |
| African American or Black | 0 | 3 | 0 |
| Asian or Pacific Islander | 7 | 5 | 7 |
| Caucasian or White | 87 | 86 | 91 |
| Native American | 7 | 0 | 0 |
| Hispanic or Latino | 13 | 2 | 3 |
| Marital status, % |  |  |  |
| Married | 67 | 61 | 64 |
| Single, never married | 27 | 34 | 31 |
| Widowed/divorced/separated | 6 | 5 | 5 |
| People in household |  |  |  |
| Mean | 2.9 | 3.3 | 3.1 |
| Percentage with children aged <18 years | 63 | 56 | 49 |
| Employed, % | 90 | 69 | 69 |
| College level education and above, % | 53 | 46 | 33 |
| Mean annual income, £ | 55,000 | 43,000 | 39,000 |
| Area of residence, % |  |  |  |
| Urban/city | 43 | 47 | 50 |
| Suburban | 40 | 44 | 32 |
| Rural | 17 | 9 | 18 |
| China | n = 30 | n = 106 | n = 43 |
| Age, years |  |  |  |
| Mean | 31 | 32 | 34 |
| 18-29, % | 40 | 37 | 21 |
| 30-39, % | 47 | 60 | 57 |
| ≥40, % | 13 | 3 | 22 |
| Ethnicity, % |  |  |  |
| African American or Black | 0 | 1 | 0 |
| Asian or Pacific Islander | 77 | 78 | 75 |
| Caucasian or White | 3 | 0 | 0 |
| Native American | 0 | 2 | 0 |
| Other | 17 | 17 | 26 |
| Marital status, % |  |  |  |
| Married | 67 | 88 | 73 |
| Single, never married | 23 | 12 | 27 |
| Widowed/divorced/separated | 10 | 0 | 0 |
| People in household |  |  |  |
| Mean | 3.0 | 3.4 | 3.7 |
| Percentage with children aged <18 years | 67 | 77 | 74 |
| Employed, % | 100 | 99 | 90 |
| College-level education and above, % | 70 | 85 | 61 |
| Mean annual income, Chinese Yen | 61 | 36 | 31 |
| Area of residence, % |  |  |  |
| Urban/city | 100 | 99 | 86 |
| Suburban | 0 | 1 | 13 |
| Rural | 0 | 0 | 1 |
| Brazil | n = 30 | n = 100 | n = 32 |
| Age, years |  |  |  |
| Mean | 33 | 31 | 31 |
| 18-29, % | 40 | 44 | 46 |
| 30-39, % | 33 | 41 | 43 |
| ≥40, % | 27 | 15 | 11 |
| Ethnicity, % |  |  |  |
| African American or Black | 17 | 12 | 17 |
| Asian or Pacific Islander | 0 | 5 | 0 |
| Caucasian or White | 73 | 74 | 67 |
| Marital status, % |  |  |  |
| Married | 84 | 65 | 57 |
| Single, never married | 13 | 33 | 39 |
| Widowed/divorced/separated | 3 | 2 | 4 |
| People in household |  |  |  |
| Mean | 3.8 | 3.7 | 3.5 |
| Percentage with children aged <18 years | 80 | 72 | 59 |
| Employed, % | 97 | 85 | 83 |
| Higher education degree, % | 77 | 63 | 50 |
| Annual income category,^a^ % |  |  |  |
| A (lowest) | 50 | 22 | 22 |
| B1 | 24 | 36 | 17 |
| B2 | 23 | 30 | 39 |
| C1, C2, D, E (highest) | 3 | 12 | 22 |
| Area of residence, % |  |  |  |
| Urban/city | 97 | 95 | 96 |
| Suburban | 0 | 4 | 0 |
| Rural | 3 | 1 | 4 |

^a^Income category cut-offs vary by country due to use of local currency values.

**Supplemental Figures**

**Supplemental Figure 1. Most important breast augmentation surgery outcomes desired by women who had undergone the procedure or were considering the procedure (n = 546).**


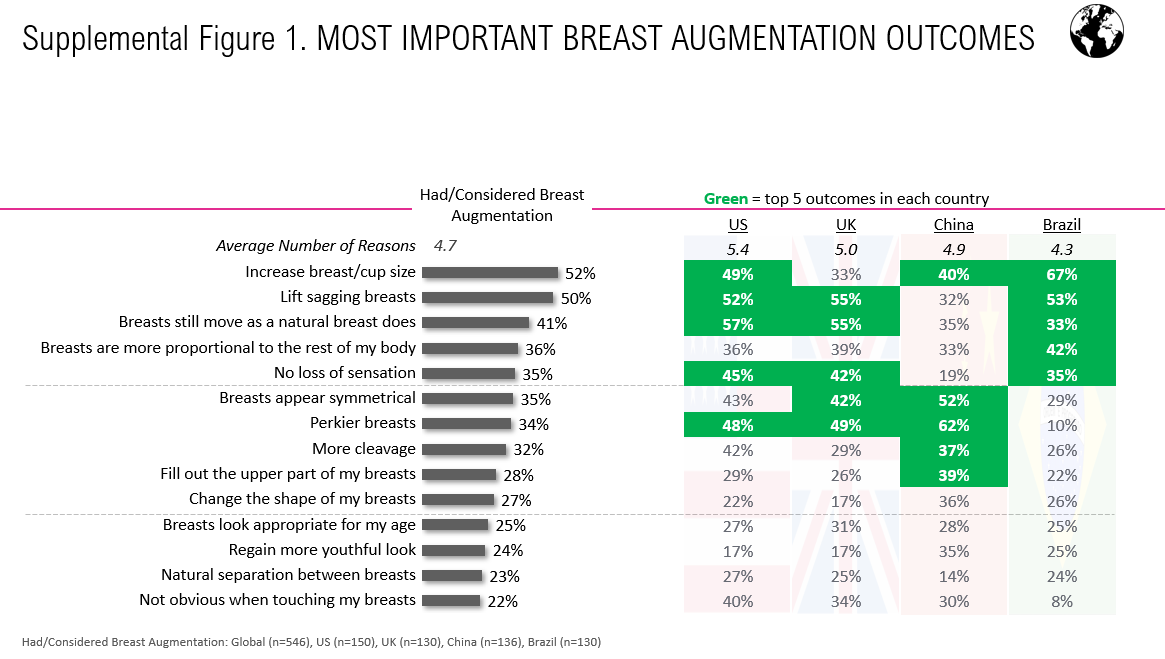


**Supplemental Figure 2. Most frequently cited personal influencer in the decision to consider breast augmentation surgery among women who had undergone the procedure or were considering the procedure (n = 546).**


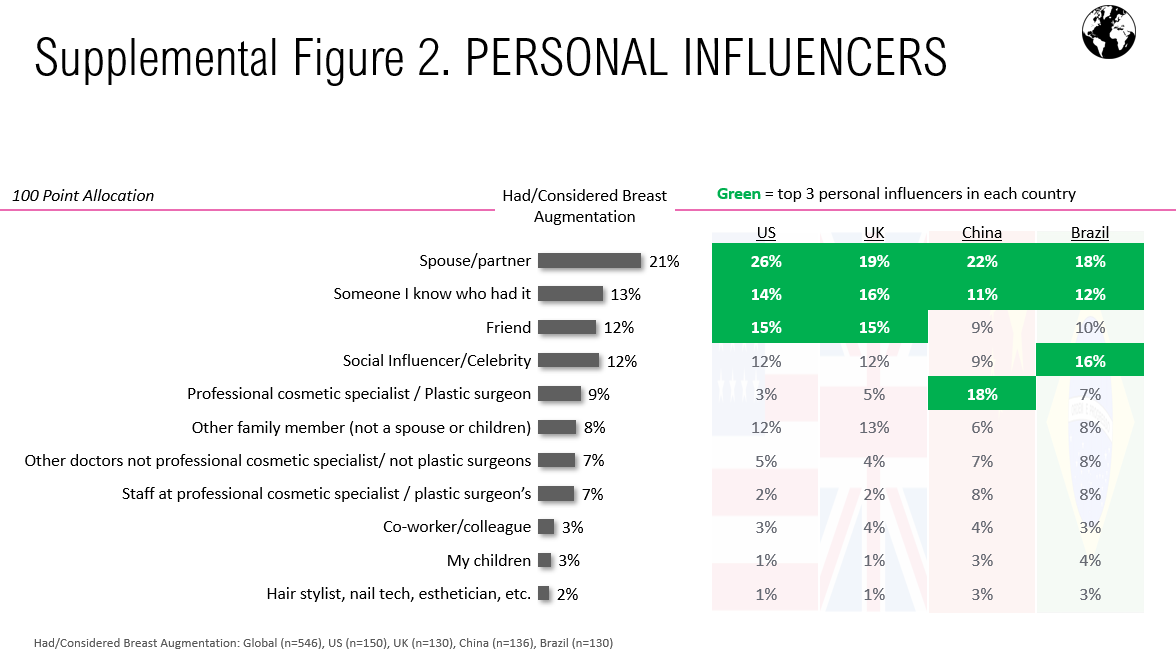


^a^The data presented reflect a 100-point allocation, with percentages summing to 100% across all identified influencers in the decision to consider breast augmentation surgery.

**Supplemental Figure 3. Most frequently cited reasons for seeking a second opinion or consulting multiple specialists among women who had either undergone the procedure or were considering breast augmentation surgery (n = 196).**


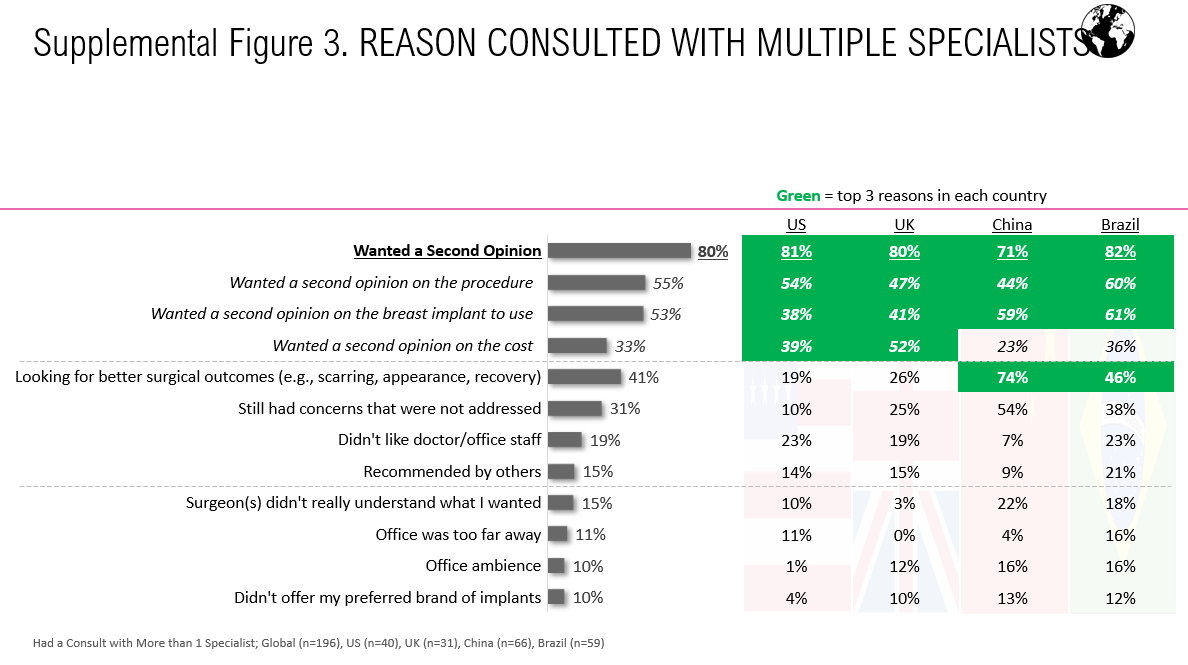


**Supplemental Figure 4. Decision-makers and influencers in choosing the breast augmentation procedure and the brand of breast implant among women who had undergone the procedure (n = 140).^a^**


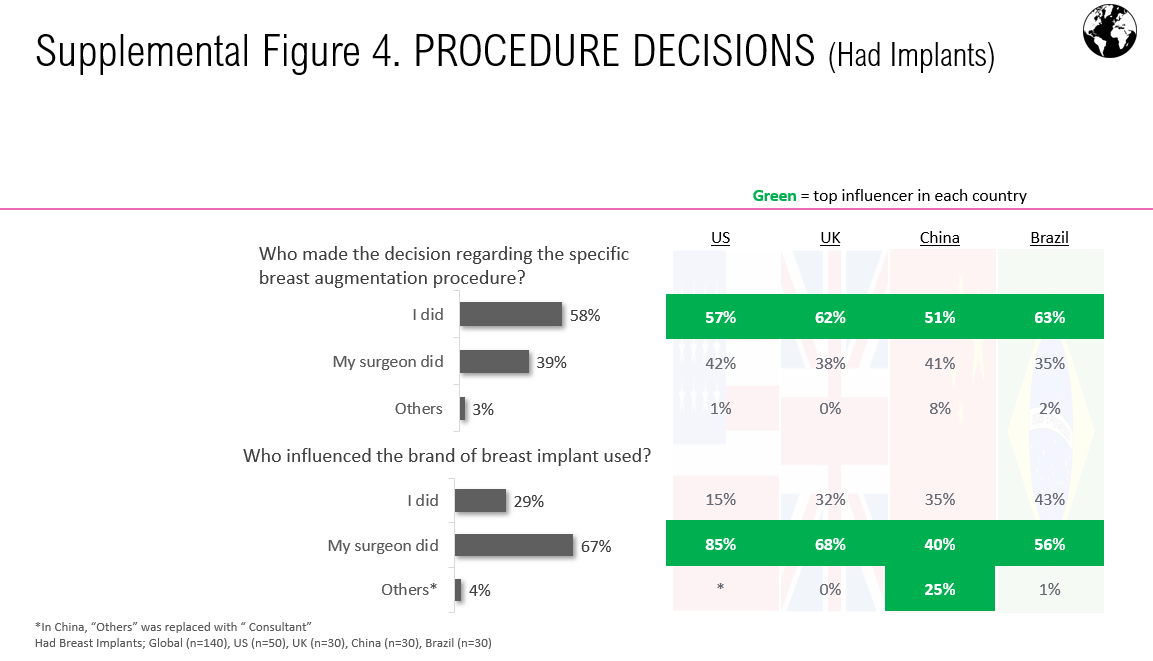


^a^In China, “Others” was replaced with “Consultant.”
